# Over-Production of the Human SLC7A10 in *E. coli* and Functional Assay in Proteoliposomes

**DOI:** 10.3390/ijms25010536

**Published:** 2023-12-30

**Authors:** Michele Galluccio, Tiziano Mazza, Mariafrancesca Scalise, Martina Tripicchio, Martina Scarpelli, Maria Tolomeo, Lorena Pochini, Cesare Indiveri

**Affiliations:** 1 Laboratory of Biochemistry, Molecular Biotechnology, and Molecular Biology, Department DiBEST (Biologia, Ecologia e Scienze della Terra), University of Calabria, Via Bucci 4C, 6C, 87036 Arcavacata di Rende, Italy; tiziano.mazza@unical.it (T.M.); mariafrancesca.scalise@unical.it (M.S.); tripicchio.martina9@gmail.com (M.T.); scarpellimartina97@gmail.com (M.S.); maria.tolomeo89@gmail.com (M.T.); lorena.pochini@unical.it (L.P.); 2 National Research Council (CNR), Institute of Biomembranes, Bioenergetics and Molecular Biotechnologies (IBIOM), Via Amendola 122/O, 70126 Bari, Italy

**Keywords:** proteoliposomes, purification, SLC, over-expression, transport, serine, amino acids

## Abstract

The human SLC7A10 transporter, also known as ASC-1, catalyzes the transport of some neutral amino acids. It is expressed in astrocytes, neurons, and adipose tissues, playing roles in learning, memory processes, and lipid metabolism, thus being involved in neurological and metabolic pathologies. Structure/function studies on this transporter are still in their infancy. In this study, we present a methodology for producing the recombinant human transporter in *E. coli.* Its transport function was assayed in proteoliposomes following the uptake of radiolabeled L-serine. After the testing of several growth conditions, the hASC-1 transporter was successfully expressed in BL21(DE3) codon plus RIL in the presence of 0.5% glucose and induced with 0.05 mM IPTG. After solubilization with C_12_E_8_ and cholesteryl hemisuccinate and purification by Ni-chelating chromatography, hASC-1 was reconstituted in proteoliposomes. In this experimental system it was able to catalyze an Na^+^-independent homologous antiport of L-serine. A Km for L-serine transport of 0.24 mM was measured. The experimental model developed in this work represents a reproducible system for the transport assay of hASC-1 in the absence of interferences. This tool will be useful to unveil unknown transport properties of hASC-1 and for testing ligands with possible application in human pharmacology.

## 1. Introduction

The human alanine serine cysteine transporter 1 (hASC-1) is the tenth member of the SLC7 family, namely SLC7A10, and mediates an Na^+^-independent antiport of neutral amino acids [1]. In the plasma membrane, hASC-1 is associated with the CD98 protein, which belongs to the SLC3 family, via a disulfide bridge between two evolutionarily conserved cysteine residues [2]. The heterodimeric assembly with CD98 is shared by other members of the SLC7 family, such as xCT (SLC7A11), LAT1 (SLC7A5), and LAT2 (SLC7A8). hASC-1 was first cloned from a brain library, where it is abundantly expressed; indeed, the transporter is present either in astrocytes or in neurons, where it contributes to the homeostasis of the neutral amino acid pools [2]. It was also reported that besides L-alanine, L-serine, and L-cysteine, hASC-1 recognizes glycine and D-serine, which are positive allosteric modulators of the N-methyl-D-aspartate (NMDA)-subtype glutamate receptors, involved in learning and memory processes [3,4]. Intriguingly, a five-times higher expression of the transporter was found in adipose tissue with respect to the brain [5]. In good agreement, a positive correlation between the adipose tissue SLC7A10 expression and circulating levels of adiponectin has been reported [6]; whereas a negative correlation between *SLC7A10* mRNA levels in subcutaneous adipose tissue and risk factors for metabolic diseases has been found [6]. Moreover, *SLC7A10* mRNA expression increased in subcutaneous adipose tissue after fat loss due to bariatric surgery, whereas reduced subcutaneous and visceral levels were found in insulin resistance [7]. A similar phenomenon has been observed in zebrafish, where the deletion of the transporter caused a strong increase in body weight and the presence of larger visceral adipocytes [7]. However, adipocytes undergoing cellular senescence are characterized by altered lipid metabolism and insulin resistance that activate feedback loops, further increasing hyperglycemia, insulin resistance, and cellular senescence [8,9]. For all these reasons, hASC-1 transporter is nowadays considered a key player in neurological disorders, metabolic diseases related to obesity, and type 2 diabetes [7]. Very recently, the *SLC7A10* gene has been proposed as a candidate for human startle disease: the G307R mutation found in patients led to structural rearrangements causing the loss of function of the transporter [10]. Moreover, the growing body of evidence linking metabolic changes to cell senescence and aging processes makes membrane transporters of nutrients, such as hASC-1, novel and under-evaluated markers for age-related diseases [9].

Therefore, the knowledge of structure/function relationships and the modulation of the hASC-1 transporter may contribute to the discovery of novel drugs for the treatment of neurological, metabolic, and age-related diseases [11,12]. In this frame, a bioinformatics approach has been previously exploited to elucidate some structural aspects: a homology model for the hASC-1 transporter was built using the structure of the bacterial transporter AdiC as a template [13]. The obtained model was used in docking and molecular dynamics studies, allowing for the identification of putative key residues for substrate binding and transport [13]. Some structure/function findings on the human ASC-1 transporter have been indirectly obtained by the comparison of structural and functional data available for the bacterial alanine–serine–cysteine exchanger (BasC) [14] with a transport assay performed in intact HeLa cells over-expressing ASC-1 [14]. A more suitable procedure for studying the transport in a single protein experimental model is the heterologous production of the human transporter and its reconstitution in proteoliposomes. This approach allows for abolishing interferences by other transport proteins and has been extensively adopted for many SLC transporters [15,16,17]. It has been particularly useful for unveiling unknown aspects of the transport mechanism and regulation of SLC25A20 (CAC), SLC1A5 (ASCT2), SLC7A5 (LAT1), SLC22A4 (OCTN1), SLC38A9, SLC7A11 (xCT) [15,18], and many others. The same procedure was revealed as suitable for drug design and testing [15]. We recently improved the production of human transporters in *E. coli* [15,19]. In this work, we investigated the best conditions for obtaining suitable expression of the human ASC-1 (hASC-1) transporter in a functionally active form. The transport assay tool will be very useful for direct studies of the structure/function relationships of hASC-1 and for ligand screening for translational medicine purposes.

## 2. Results

### 2.1. Expression in E. coli and Purification of hASC-1 Transporter

**Figure 1:**
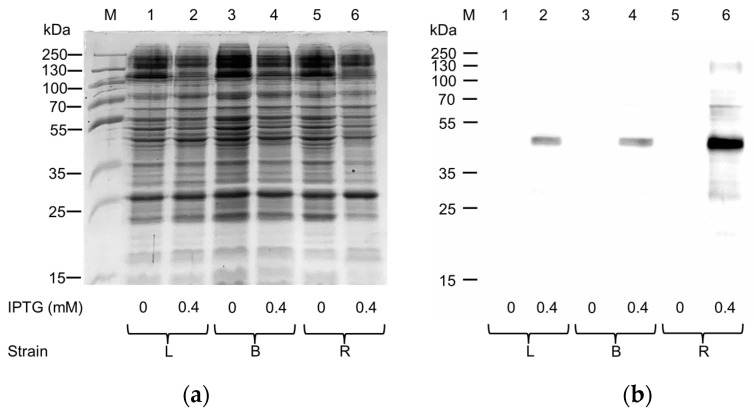
Expression of hASC-1 transporter in different *E. coli* strains: L, Lemo21(DE3); B, BL21(DE3) codon plus RIL; R, Rosetta(DE3). (**a**) SDS-PAGE of insoluble fraction from uninduced (1, 3, and 5) and induced (2, 4, and 6) cell lysates; M, page ruler prestained plus marker. The gel was stained using Coomassie blue staining. (**b**) Western blotting using anti-His antibody of the samples loaded as in (**a**).

The human ASC-1-pH6EX3 construct was exploited to transform Lemo21(DE3), Rosetta(DE3), and BL21(DE3) codon plus RIL *E. coli* strains in order to attempt protein expression. A single colony from each plate was inoculated and cultivated overnight in a specific volume of LB broth. The following day, a 1:20 dilution was added to fresh LB broth with antibiotics, and the cultures (six flasks) were kept at 37 °C until they reached the exponential phase of growth (OD~0.8). Then, the temperature was lowered to 28 °C, and 0.4 mM IPTG was added to induce protein expression for 4 h according to the conditions already tested by our group for the expression of another member of the SLC7 family, namely LAT1 [15]. Even though no apparent differences were observed following SDS-PAGE analysis (Figure 1a) between the uninduced and induced samples, the Western blotting analysis showed a specific reaction exclusively in the insoluble fraction (pellet) of induced cell lysate from any of the tested *E. coli* strains (Figure 1b). The strongest signal was obtained in the pellet fraction of the induced Rosetta culture (Figure 1b, Lane 6).

**Figure 2:**
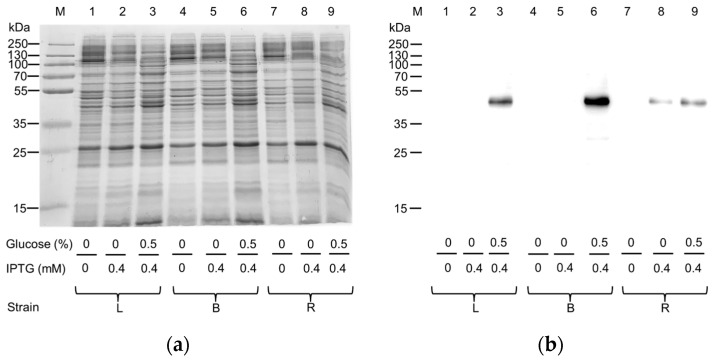
Effect of glucose addition on the expression of hASC-1 transporter in different *E. coli* strains: L, Lemo21(DE3); B, BL21(DE3) codon plus RIL; R, Rosetta(DE3). (**a**) SDS-PAGE of insoluble fraction from uninduced (1, 4, and 7) and induced cell lysates, in the absence (2, 5, and 8) or presence of 0.5% glucose (3, 6, and 9); M, page ruler prestained plus marker. The gel was stained using Coomassie blue staining. (**b**) Western blotting using anti His antibody of the samples loaded as in (**a**).

To improve the production of the protein of interest, the same *E. coli* strains were cultured either in the absence or in the presence of 0.5% glucose (Figure 2), since it is known that glucose addition may positively affect the production of human plasma membrane proteins [15,16].

**Figure 3:**
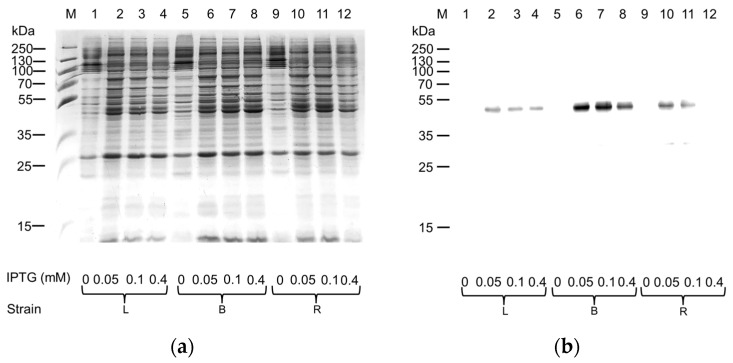
Effect of the IPTG concentration on the expression of the hASC-1 transporter in different *E. coli* strains: L, Lemo21(DE3); B, BL21(DE3) codon plus RIL; R, Rosetta(DE3). (**a**) SDS-PAGE of insoluble fraction from uninduced (1, 5, and 9) and induced cell lysates, grown in the presence of 0.5% glucose and IPTG 0.05 mM (2, 6, and 10), 0.1 mM (3, 7, and 11), 0.4 mM (4, 8, and 12); M, page ruler prestained plus marker. The gel was stained using Coomassie blue staining. (**b**) Western blotting using anti-His antibody of the samples loaded as in (**a**).

Despite glucose addition, no clear improvement in production was observed after SDS-PAGE analysis (Figure 2a). Interestingly, the Western blotting analysis on the one hand confirmed Rosetta as the most effective strain in the absence of glucose (Figure 2b, Lane 8), and on the other hand highlighted that the amount of hASC-1 produced was maximal in the BL21 strain cultured in the presence of glucose (Figure 2b, Lane 6). Since we had already observed that the decrease in IPTG concentration could trigger an increase in the production of other SLC transporters from the same [16] or other SLC families [19], we tested three different IPTG concentrations: 0.05 mM, 0.1 mM, and 0.4 mM (Figure 3).

**Figure 4:**
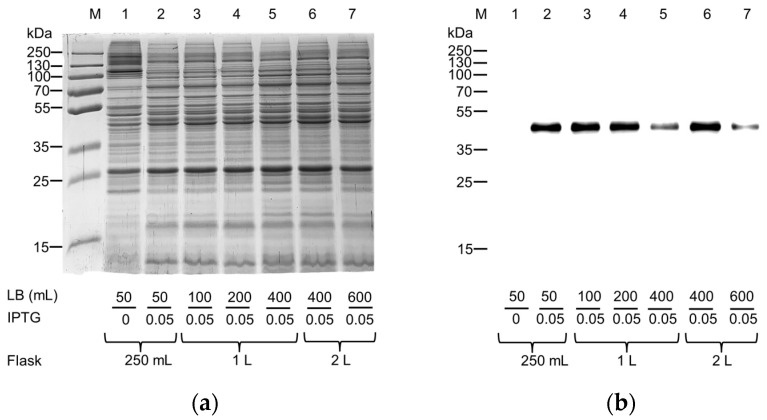
Effect of the oxygen on the expression of hASC-1 transporter in *E. coli* BL21(DE3) codon plus RIL. (**a**) SDS-PAGE of insoluble fraction from uninduced (Lane 1) and induced cell lysates (Lanes 2–7), grown in the presence of 0.5% glucose and IPTG 0.05 mM in different Erlenmeyer flasks filled as indicated in the figure. The gel was stained using Coomassie blue staining. (**b**) Western blotting using anti-His antibody of the samples loaded as in (**a**).

Decreasing IPTG concentration had a positive effect on protein production in all the used *E. coli* strains, allowing us to observe a difference among the pellets of induced and uninduced cell lysates following SDS-PAGE analysis (Figure 3a). The Western blotting analysis confirmed that the most suitable strain for the production of hASC-1 was the BL21(DE3) codon plus RIL cultured in the presence of 0.05–0.1 mM IPTG. Since it is known that oxygen availability strongly influenced the production of the hxCT protein [16], this parameter was also tested on the production of the protein of interest by modifying the ratio of LB broth (mL) to the flask capacity (Figure 4).

**Figure 5:**
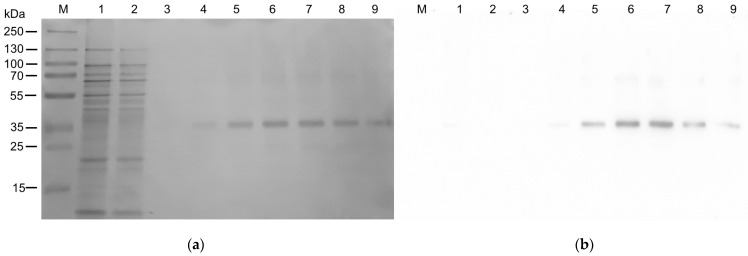
Purification of hASC-1 transporter. (**a**) The protein was solubilized in 10% (*w*/*v*) C_12_E_8_: 2% (*w*/*v*) CHS and purified by affinity chromatography. SDS-PAGE of the following fractions: (Lane 1), flow through fraction containing the unbound proteins; (Lane 2), fraction of the proteins eluted with washing buffer; (Lanes 3 to 9), fractions of the protein eluted with washing buffer supplemented with 500 mM imidazole. The gel was stained using Coomassie blue staining. (**b**) Western blotting using anti-His antibody of the samples loaded as in (**a**).

Interestingly, from this experiment, it was observed that the capacity of the flask should be at least five times greater (i.e., increased oxygen volume) than the cultured broth volume (Figure 4b Lanes 2–4, 6) for improving the hASC-1 production. Then, starting from 24 mL of bacterial lysate obtained as described above, the protein of interest was purified by nickel chelating affinity chromatography using the on-column refolding approach (Figure 5). The solubilization of hASC-1 was performed using the non-ionic detergent C_12_E_8_ combined with cholesteryl hemisuccinate (CHS) in a ratio of 5:1, which was revealed as useful in previously published data [20]. To promote the interaction between the 6His tag of the hASC-1 and the Ni^2+^ ions of the resin, the protein was denatured in the presence of the chaotropic agent urea and the disulfide-reducing reagent 1,4-Dithioerythritol (DTE). Interestingly, even though a mild detergent was used, more than 90% of the hASC-1 was solubilized, as shown by SDS-PAGE and Western blotting analysis (Supplementary Figure S1).

Then, the solubilized protein was applied on an Ni^2+^ affinity column for the previously mentioned on-column refolding. In this procedure, the denaturing agent urea is completely removed and the concentration of C_12_E_8_ is slowly reduced, maintaining the 5:1 ratio with CHS. The SDS-PAGE analysis of Figure 5 clearly shows that hASC-1 was purified using an elution buffer containing imidazole. From the described purification protocol, a yield of 0.5 mg hASC-1 per liter of culture was obtained. This value is in the range of previously set-up procedures for other SLC family members [16,21]. The homogeneity of the purified hASC-1 was evaluated by SEC analysis using the same C_12_E_8_:CHS buffer used for the elution (Supplementary Figure S2). The analysis showed a major symmetric peak corresponding to a homogeneous protein fraction.

### 2.2. Reconstitution in Proteoliposomes of hASC-1

**Figure 6:**
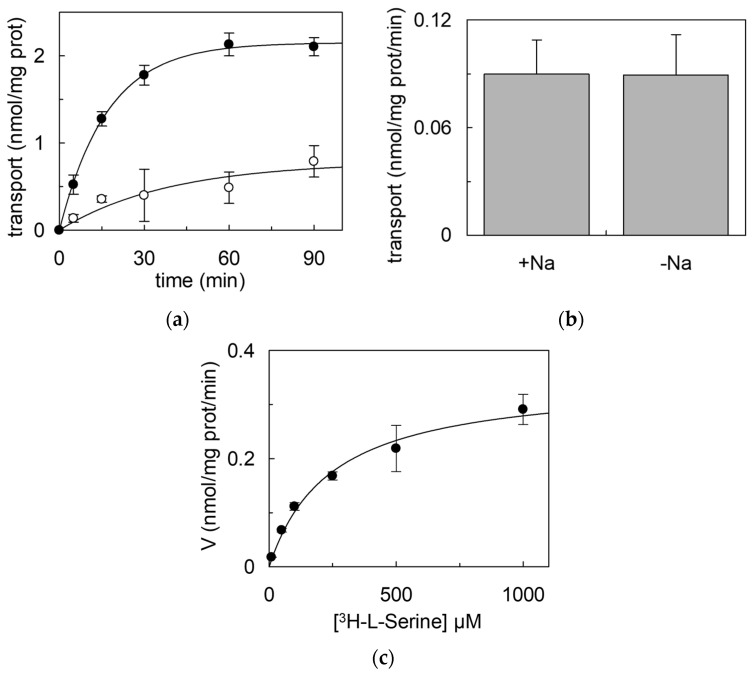
[^3^H]-L-serine uptake in proteoliposomes. hASC-1 was purified and reconstituted in proteoliposomes, as described in Section 4. (**a**) Transport was started by the addition of 100 µM [^3^H]-L-serine to proteoliposomes containing 10 mM L-serine (●) or without internal substrate (○). The transport was stopped at the indicated times as described in Section 4. (**b**) Dependence of the transport rate of hASC-1 reconstituted in proteoliposomes on external Na^+^. The transport was started by the addition of 100 µM [^3^H]-L-serine in the absence or in presence of 50 mM external Na^+^-gluconate to proteoliposomes containing 10 mM internal L-serine. The transport was measured after 30 min. Statistical analysis was performed by Student’s *t*-test, with *p* < 0.05 indicating significant difference. (**c**) Kinetics of hASC-1 reconstituted in proteoliposomes. The transport was started by the addition of indicated concentrations of [^3^H]-L-serine to proteoliposomes containing 10 mM L-serine. The transport was measured after 5 min, as described in Section 4. Data were analyzed according to the Michaelis–Menten equation as transport rate versus [^3^H]-L-serine concentration. In (**a**–**c**), results are means ± SD from three independent experiments.

To evaluate the functionality of the recombinant hASC-1, the purified protein was subjected to a desalting on-column treatment to remove imidazole and then reconstituted in artificial vesicles, i.e., proteoliposomes. The hASC-1 reconstitution was performed using liposomes prepared with phosphatidylcholine and cholesterol with a protein:lipid ratio of 0.005:10 (mg/mg). In this single-protein tool, a functional characterization was performed in the absence of interferences deriving from other cell proteins. The transport activity was measured as the uptake of extraliposomal [^3^H]-L-serine in the presence or absence of the internal counter-substrate. Interestingly, and in line with literature data, the transporter worked as an exchanger; indeed, only in the presence of intraliposomal L-serine a significant uptake of [^3^H]-L-serine was measured (Figure 6a and Supplementary Figure S3). The exchange occurred at physiological pH and was Na^+^-independent (Figure 6b), in good agreement with previous reports [1,2]. In the same experimental system, kinetics was also evaluated, and the external Km and the Vmax for L-serine transport were derived, being 242.3 ± 41.0 µM and 0.347 ± 0.040 nmol/mg protein/min, respectively (Figure 6c). The transport activity of SEC-eluted hASC-1 (Supplementary Figure S4) was very similar to that of the PD-10 desalted protein (Figure 6).

## 3. Discussion

### 3.1. Expression of ASC-1

In the present work, a procedure is presented for studying the hASC-1 transporter reconstituted in proteoliposomes. This experimental tool is suitable for unveiling novel aspects of the structure/function relationships of membrane transporters in the absence of interferences by other transport systems or enzymes that, on the contrary, are present in intact cell systems. Moreover, the low protein/lipid ratio of proteoliposomes results in a relatively long time course for achieving the equilibrium of external/internal [^3^H]-L-serine with optimal kinetic measurement (Figure 6a) [22]. For properly assembling the proteoliposome vesicles with the sole transport protein of interest, the first crucial step is the over-expression of the protein followed by its purification. It has to be stressed that the best expression conditions cannot be established a priori; indeed, for obtaining the heterologous expression of the hASC-1 transporter, several *E. coli* strains with different molecular features have been cultured and tested. Interestingly, the BL21(DE3) codon plus RIL cells, which supply tRNA specific for human arginine, isoleucine, and leucine codons, were revealed to be the most proficient in hASC-1 expression. Some recently introduced changes with respect to standard procedures for bacterial over-expression have been combined for hASC-1. An IPTG concentration much lower than the standard ones, was used, as for over-expressed SLCs in recent studies [15,19]. This, together with glucose addition, exerted a positive effect on protein production, probably due to catabolite repression, which prevented the production of the protein in the absence of an inducer, avoiding toxicity problems [15]. Indeed, a putative toxic effect of the recombinant protein was confirmed by the inverse relationship between the inducer concentration and protein synthesis, as already observed for other amino acid transporters, probably due to Sec-translocon saturation [16,19]. A fundamental role in hASC-1 production was exerted by oxygen availability, which was increased by decreasing the broth volume in each flask. In this respect, hASC-1 behaved, in *E. coli*, similarly to another member of the SLC7 family, the SLC7A11 (xCT) transporter [16]. The dependence on oxygen may be linked to a metabolic load affecting *E. coli* cells, which positively respond to the increased oxygen availability for overcoming the stress due to the over-expression of a potentially toxic transporter. Furthermore, it cannot be excluded that the presence of oxygen allowed *E. coli* to produce the human transporter in a condition from which the refolding was easier to perform. Indeed, this was different from other human SLC transporters produced in *E. coli*, which were refolded on-column, substituting the ionic detergent sarkosyl with a non-ionic detergent [16,21]. hASC-1 could be solubilized directly with the non-ionic C_12_E_8_. To facilitate the solubilization with this detergent, CHS was added during the solubilization procedure, as previously carried out to improve solubilization or stability [23,24,25,26] or to maximize the purification efficiency of other transporters and channels [27,28,29,30]. In particular, the addition of CHS has been shown to significantly enhance the stability of eukaryotic proteins by mimicking the interaction with cholesterol [31,32]. The addition of CHS also influences the size of detergent micelles, potentially affecting the protein extraction and purification processes [27,33]. Thus, we can hypothesize that CHS added during the solubilization may have a positive impact on the transport activity, probably due to a better refolding or to a direct effect on the transporter activity, as previously observed for other transporters, such as hOCTN1, hASCT2, and CAT2 from *S. lycopersicum* [15,34]. The solubilization by a non-ionic detergent in the presence of CHS represents, to our knowledge, an important novelty for SLC transporters expressed in bacteria. The influence of CHS or cholesterol or other lipids on the structure/function relationships, including the oligomerization state, of this transporter, is in the course of investigation. This is, indeed, an open field, considering the growing body of evidence showing that the membrane composition affects protein stability and function, not only for the chemo-physical properties of the cell membranes, such as fluidity and curvature, but because of the physical interactions occurring between some specific lipids with hydrophobic portions of the membrane proteins [35,36].

### 3.2. Reconstitution of ASC-1

Not surprisingly, the purified hASC-1 showed a significant transport activity in proteoliposomes, allowing us to hypothesize that the ancillary protein CD98 would not be necessary for intrinsic transport function, as was previously demonstrated for LAT1 and xCT, belonging to the same family [2,16]. However, at this stage, we cannot exclude that the CD98 subunit may play a role in the regulation of hASC-1 substrate specificity/affinity, subcellular localization, and response to physiological modulators. Indeed, as stated in the introduction, the knowledge of the physiological and pathological roles of this transporter is still puzzling. Notwithstanding, a key function in maintaining homeostasis of amino acids is expected in crucial body districts, such as the brain, where ASC-1 dysfunctions may underlie some features of age-related conditions and neurological disorders, such as memory loss. In this frame, the present work represents a first step towards setting up a suitable model for studying hASC-1 function, defining the transport mechanism and structure/function relationships. Indeed, the description of basic transport features, i.e., counter-exchange of amino acids, micromolar Km for the external substrate, and Na^+^-independence from the external side, strongly indicates that hASC-1 works in liposomes with similar features to those described in intact cells [1,2]. Importantly, given the previously cited advantages of the proteoliposome experimental model, it will be possible to define the functional properties of hASC-1, such as the mechanism of transport of D-amino acids, which is not yet defined.

## 4. Materials and Methods

Protease inhibitor cocktail (P8849), Monoclonal Anti-polyHistidine-Peroxidase antibody (A7058), Amberlite XAD-4 (06444), egg yolk phospholipids, cholesterol, cholesteryl hemisuccinate (CHS), Sephadex G-75 (G75120), L-serine from Merck Life Science KGaA, Darmstadt, Germany; PD-10 desalting columns (17-0851-01), HisTRAP HP Ni^2+^ chelating columns (17524801), Superose 6 Increase 10/300 GL (29091596) from Cytiva, Milan, Italy; Isopropyl β-D-thiogalactopyranoside (IPTG, A4773) and Coomassie^®^ Brilliant Blue R-250 from AppliChem, Milan, Italy; restriction endonucleases, T4 DNA ligase (EL0014), and Phusion™ High-Fidelity DNA Polymerase (F530S) from Thermo Fisher scientific; *E. coli* Rosetta(DE3) strain from Novagen, Milan, Italy; MegaMan Human Transcriptome Library, BL21(DE3) codon plus RIL strain from Agilent technologies, Milan, Italy; Lemo21(DE3) strain from New England Biolabs, Ipswich, MA, USA. L-[^3^H]-serine from Perkin Elmer, Waltham, MA, USA; C_12_E_8_ from TCI Europe, Zwijndrecht, Belgium. 

### 4.1. Cloning of Human hASC-1

The cDNA encoding for human ASC-1 transporter (SLC7A10) (UniProtKB: Q9NS82; GenPept accession no. NP_062823.1) was amplified from MegaMan Human Transcriptome Library using the following specific primers: 5′-CCGGAATTCTATGGCCGGCCACACGCAGCAG-3′ (forward) and 5′-CCGCTCGAGTCATTGTGGCTTCGAGGGCTT-3′ (reverse), respectively. The amplified sequence was cloned between EcoRI and XhoI restriction sites of the pH6EX3 expression vector. The resulting recombinant plasmid, defined as pH6EX3-hASC-1, encodes a 6His-tagged fusion protein corresponding to the hASC-1 carrying an extra N-terminal MSPIHHHHHHLVPRGSEASNS sequence.

### 4.2. Expression of Human ASC-1 Transporter

To produce the 6His-hASC-1 recombinant protein, *E. coli* BL21(DE3) codon plus, Rosetta(DE3), and Lemo21(DE3) cells were transformed with the pH6EX3-hASC-1 construct and selected on LB-agar plates supplemented with 100 µg/mL ampicillin and 34 µg/mL chloramphenicol. Different media (LB, TY 2X, or Terrific) prepared in the absence or presence of 0.5% (*w*/*v*) glucose were tested. For small-scale experiments, one colony (one for each strain) was inoculated in 5 mL of a specific medium and cultured overnight at 37 °C under rotary shaking (160 rpm). The following day, two aliquots of 2.5 mL each were diluted 1:20 in fresh medium supplemented with the specific antibiotics and glucose where indicated, and specifically treated. When the optical density measured at 600 nm wavelength was about 0.8–1, the growth temperature was lowered to 28 °C, and different IPTG concentrations (from 0.05 to 0.4 mM) were tested to induce protein expression for up to 6 h, except for one aliquot grown without inducer (negative control). Every two hours, aliquots were collected and centrifuged at 6000× *g* at 4 °C for 10 min; the insoluble fractions (pellets) were stored at −20 °C.

#### Effect of O_2_ on Protein Expression

One colony was inoculated in 100 mL of LB broth and cultured overnight at 37 °C in the presence of 0.5% (*w*/*v*) glucose and the required antibiotics. The day after, several aliquots were diluted 1:20 in fresh medium, and antibiotics and glucose were added, filling Erlenmeyer flasks of the same capacity to different levels, as previously carried out for another member of the SLC7 family [16]. At the mid-logarithmic phase of growth, 0.05 mM IPTG was added to induce the protein synthesis for up to 6 h at 28 °C. To monitor cell growth and protein production, aliquots were collected every two hours, centrifuged, and stored as previously indicated.

### 4.3. Protein Quantification and Analysis

A bacterial pellet aliquot, after thawing, was dissolved in a resuspension buffer (20 mM Hepes/Tris, 300 mM NaCl pH 7.5) containing protease inhibitor cocktail according to manufacturer instructions. The volume of resuspension buffer was calculated proportionally to the OD measured at 600 nm wavelength: about 20 mL for a pellet deriving from 400 mL of cell culture. The bacterial suspensions were sonified in pulses, in an ice bath, for 5 min (1 s on, and 1 s off) using a Branson SFX 550 sonifier (Emerson, Round Rock, TX, USA) at 30%. The cell lysate was centrifuged at 13,000× *g* and the pellet obtained was stored at −20 °C or further analyzed. Protein concentration was measured by the Lowry method, modified for the presence of detergents [37]. Proteins were separated by 12% (*w*/*v*) SDS–PAGE according to Laemmli [38] using the Hoefer SE260 mini-vertical unit and stained with Coomassie Brilliant Blue. Quantitative evaluation of Coomassie-stained protein bands was carried out using the Chemidoc imaging system equipped with Image Lab 6.1 software (Bio-Rad, Hercules, CA, USA).

### 4.4. Purification of the hASC-1 Protein

To purify 6His- hASC-1 protein, the pellet deriving from 24 mL bacterial suspension was washed twice with 0.1 M Tris/HCl pH 8.0 and centrifuged at 13,000× *g* for 10 min. The new pellet containing the insoluble protein fraction was solubilized by the addition of 8 mL of 8 M urea (4 M final concentration), 400 µL of 500 mM (14 mM final concentration) DTE, 1080 µL of 10% (*w*/*v*) C_12_E_8_: 2% (*w*/*v*) CHS corresponding to a C_12_E_8_/CHS ratio of 5:1 (*w*/*w*), and 500 µL 200 mM L-serine, and mixed for 30 min in a fixed angle rotator for tubes. Then, 5 mL of a buffer containing: 0.1% (*w*/*v*) C_12_E_8_: 0.02% (*w*/*v*) CHS, 200 mM NaCl, 10% (*w*/*v*) glycerol, 10 mM serine, 3 mM DTE, and 20 mM Tris/HCl at pH 8.0 was added and the rotation continued for 30 min in the same conditions. Then, the sample was centrifuged (15,000× *g*, 10 min, 4 °C), and the supernatant was applied onto a Ni^2+^ affinity gel column His-TRAP HP (CV 5 mL on Akta start-Cytiva system) equilibrated with 3 CV of RB. Then, the column was washed with 3 CV of a buffer containing 0.1% (*w*/*v*) C_12_E_8_: 0.02% (*w*/*v*) CHS, 200 mM NaCl, 10% glycerol, 1 mM L-serine, 3 mM DTE, and 20 mM Tris/HCl at pH 8.0 to remove unbound proteins. A second washing step was performed with 2 CV of washing buffer (0.03% (*w*/*v*) C_12_E_8_: 0.006% (*w*/*v*) CHS, 200 mM NaCl, 10% (*w*/*v*) glycerol, 1 mM L-serine, 3 mM DTE, 20 mM Tris/HCl at pH 8.0, and 50 mM imidazole). Then, the protein was eluted using 3 CV of elution buffer composed of: 0.03% (*w*/*v*) C_12_E_8_: 0.006% (*w*/*v*) CHS, 200 mM NaCl, 10% (*w*/*v*) glycerol, 1 mM L-serine, 3 mM DTE, 20 mM Tris/HCl at pH 8.0, and 500 mM imidazole. Fractions of 1 mL corresponding to the elution peak on Akta start were collected and pulled together for the following purification step. The purified hASC-1 protein was present in fractions 6–8. The fractions were pulled together and passed through a PD-10 column to remove imidazole, using a buffer composed of 0.03% (*w*/*v*) C_12_E_8_: 0.006% (*w*/*v*) CHS, 10% (*w*/*v*) glycerol, 1 mM L-serine, 3 mM DTE, and 20 mM Tris/HCl at pH 8.0. The protein was then used for reconstitution in proteoliposomes, as described in the following paragraph. Fractions containing hASC-1 (Figure 5a, Lanes 6–8) were pulled together and applied onto a Superose 6 Increase 10/300 GL gel filtration column (Cytiva) equilibrated with SEC buffer (20 mM Tris/HCl pH 8.0, 200 mM NaCl,1 mM Serine, 3 mM DTE, 0.03% C_12_E_8_ 0.006% CHS).

### 4.5. Reconstitution of hASC-1 in Proteoliposomes

The desalted hASC-1 was used for reconstitution in proteoliposomes. The composition of the initial mixture was: 5 µg of the purified protein in desalting buffer, 100 µL of 10% (*w*/*v*) C_12_E_8_, 100 µL of 10% (*w*/*v*) egg yolk phospholipids in the form of sonicated liposomes prepared with 7.5% cholesterol as previously described [18], 10 mM L-serine (or no addition as indicated in figure legends), 10 mM DTE, and 20 mM Hepes/Tris pH 7.5, in a final volume of 700 µL. The mixture was then incubated for 40 min under rotatory stirring (1200 rpm) at 23 °C with 0.5 g of Amberlite XAD-4, for detergent removal from mixed micelles.

### 4.6. Transport Measurements in Proteoliposomes Reconstituted with hASC-1

To remove the external compounds, 600 µL of proteoliposomes derived from the reconstitution procedure was passed through a Sephadex G-75 column (0.7 cm diameter × 15 cm height) equilibrated with a buffer containing 20 mM Hepes/Tris pH 7.5. The eluate was aliquoted into 100 µL samples for transport assay. The uptake was started by adding 100 µM [^3^H]-L-serine (20 Ci/mmol with a final radioactivity of 0.25 µCi in each 100 µL sample) at room temperature and stopped, at the desired times, as indicated in the figure legends. To remove the external radioactivity, 100 µL of each sample was passed through a Sephadex G-75 column (0.6 diameter × 8 cm height), buffered with 50 mM NaCl. Liposomes were eluted with 1 mL of 50 mM NaCl and added to a 3 mL of scintillation mixture for radioactivity counting. In control samples, the desalting buffer was used in place of protein. The experimental values were analyzed by the subtraction of each sample from the respective control. For kinetics evaluation, different concentrations of [^3^H]-L-serine were added during the transport assay, as described in the figure legend. Data from time course analysis and kinetics were plotted according to the first-order rate equation and Michaelis Menten equation, respectively. The experimental data were analyzed using the software Grafit 5.0.13 version.

### 4.7. Western Blotting

Recombinant hASC-1 protein was immuno-detected using the Monoclonal Anti-polyHistidine-Peroxidase antibody 1:10,000 after 1 h incubation at room temperature. The reaction was detected by Electro Chemi Luminescence (ECL).

## Data Availability

The data presented in this study are available on request from the corresponding author.

## Supplementary Materials

The supporting information can be downloaded at: https://www.mdpi.com/article/10.3390/ijms25010536/s1.
