# Supplementary material for: Over-Production of the Human SLC7A10 in E. coli and Functional Assay in Proteoliposomes
Source: Int J Mol Sci. 2023 Dec 30;25(1):536. doi: 10.3390/ijms25010536 (PMC10779382; doi:10.3390/ijms25010536)
Supplement: Supplementary file 1 [file ijms-25-00536-s001.zip › ijms-2775974-supplementary.pdf]

Article

Over-Production of The Human SLC7A10 in *E. coli* and Functional Assay in Proteoliposomes

Michele Galluccio[1a], Tiziano Mazza[1a], Mariafrancesca Scalise[1], Martina Tripicchio[1], Martina Scarpelli[1], Maria Tolomeo[1], Lorena Pochini[1,2], and Cesare Indiveri [1,2,*]**

1 Department DiBEST (Biologia, Ecologia e Scienze della Terra) Laboratory of Biochemistry, Molecular Biotechnology, and Molecular Biology, University of Calabria, Via Bucci 4C, 6C, 87036 Arcavacata di Rende, Italy; michele.galluccio@unical.it; tiziano.mazza@unical.it; mariafrancesca.scalise@unical.it; tripicchio.martina9@gmail.com; scarpellimartina97@gmail.com; maria.tolomeo89@gmail.com

2 National Research Council (CNR), Institute of Biomembranes, Bioenergetics and Molecular Biotechnologies (IBIOM), via Amendola 122/O, 70126 Bari, Italy; cesare.indiveri@unical.it

* Correspondence: Department DiBEST (Biologia, Ecologia e Scienze della Terra), University of Calabria, Via P. Bucci cubo 4C, 87036 Arcavacata di Rende (CS), Italy. Tel:+39-0984-492939. Fax: +39-0984-492911. cesare.indiveri@unical.it

** Co-corresponding author. michele.galluccio@unical.it

a These authors contributed equally to this work.

Supplementary Materials: The following supporting information can be downloaded at: www.mdpi.com/article/10.3390/ijms25010536/s1, Figure S1-S3.

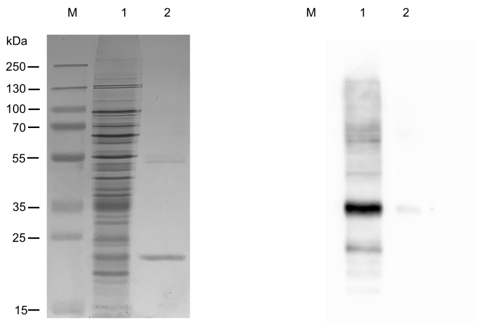

(a) **(b)**

Supplementary Figure S1. Solubilization of the hASC-1 transporter. (**a**) SDS-PAGE of soluble (lane 1) and insoluble fraction (pellet, lane 2) after solubilization procedure as described in 4.4; M, page ruler prestained plus marker. The gel was stained using Coomassie Blue staining. (**b**) Western blotting using anti His antibody of the samples loaded as in (**a**).

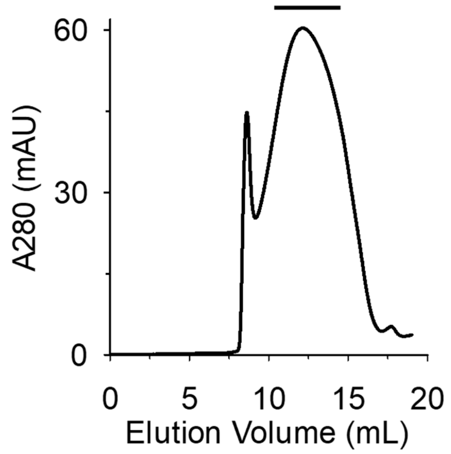

Supplementary Figure S2. Size-exclusion chromatography profile of hASC1. The SEC analysis was conducted as described in Materials and Methods; black bar indicates the fractions used for functional analysis representing the 92.33% of the total peaks area.

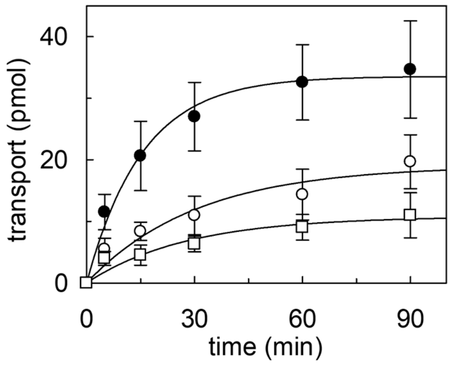

Supplementary Figure S3. [^{3}H]-L-serine uptake in proteoliposomes. The transport was started by adding 100 μM [^{3}H]-L-serine to proteoliposomes containing 10 mM L-serine (●) or without internal substrate (○) or to controls (□) i.e., liposomes without reconstituted protein. The transport was stopped at the indicated times as described in Materials and Methods. Results are means ± SD from three independent experiments.

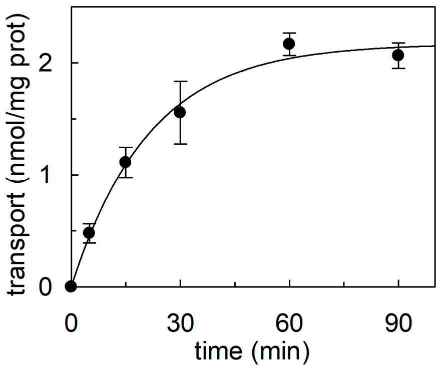

Supplementary Figure S4. [^{3}H]-L-serine uptake in proteoliposomes. The transport was started by adding 100 µM [^{3}H]-L-serine to proteoliposomes containing 10 mM L-serine prepared with protein eluted from SEC column. The transport was stopped at the indicated times as described in Materials and Methods. Results are means ± SD from three independent experiments.
